# A coordinated approach to mapping neglected tropical diseases

**Published:** 2013

**Authors:** Els Mathieu, Alaine Knipes

**Affiliations:** Resident Advisor: Central African Field and Applied Epidemiology Training Program, Centers for Disease Control and Prevention, Yaounde, Cameroon.; Guest Researcher: Field Epidemiology Training Program, Centers for Disease Control and Prevention, Atlanta, Georgia, USA

**Figure:**
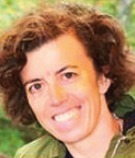
Els Mathieu

**Figure:**
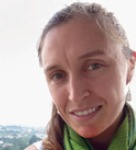
Alaine Knipes

Disease prevalence mapping allows countries to identify infected individuals and populations in need of disease-control measures, such as mass drug administration (MDA) with preventive chemotherapy. For countries with unlimited time and resources, disease-specific prevalence mapping may be carried out in a slow, careful manner, by large teams of diagnostic technicians, supervisors, and drivers. These mapping protocols, established by the World Health Organization (WHO), are intended to be carried out independently, by each respective disease-control programme, in order to assess prevalence of neglected tropical diseases (NTDs) in individual populations. They work particularly well in areas endemic for one NTD.

In reality, the countries most affected by NTDs have limited resources for mapping, and are endemic for two or more NTDs, for example lymphatic filariasis (LF), and/or trachoma, and/or schistosomiasis and/or soil transmitted helminths (STH). For these countries, with efficiency and field-practicality in mind, the coordinated threshold mapping (CTM) method was developed. The method enables coordinators from two or more disease programmes to work together to determine the prevalence of two or more diseases in a population, at one time. The CTM method achieves the same goals as the WHO's disease-specific protocols, namely identifying the need for MDA with preventive chemotherapy. It does not provide prevalence figures, but instead aims to determine whether a disease has attained the threshold necessitating a public health intervention.^1^ Therefore, rather than carrying out simultaneous, independent mapping efforts in areas endemic for more than two NTDs, at a high cost to the national control programmes, the CTM method saves countries precious time (smaller sampling size) and resources (fewer survey team members). Table 1 gives a comparison of disease-specific prevalence mapping and CTM.

**Figure:**
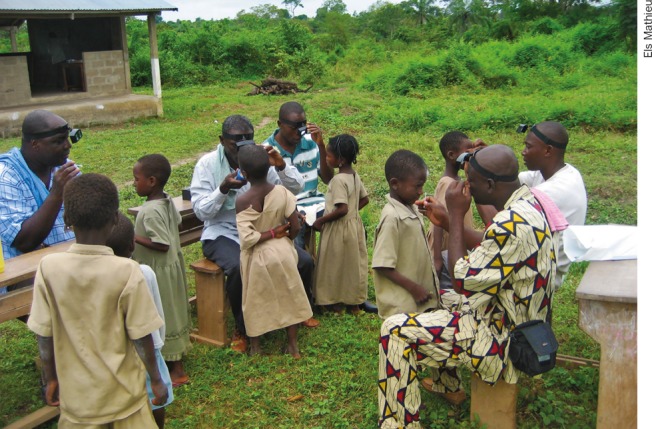
After giving stool and urine samples (to detect soil-transmitted helminths and schistosomiasis, respectively) the children are checked for signs of trachoma. TOGO

It is the responsibility of a national-level coordinator to work together with the coordinators of the various NTD control programmes to compile historic data that will determine areas where NTDs are suspected to be endemic. Once the areas in need of NTD prevalence mapping have been identified, a protocol is agreed upon at the national level. In the CTM method, each disease module uses the WHO-recommended standard indicators and diagnostic methods (Table 2), and is field-tested and independent. As such, a protocol is built to suit the particular mapping needs of a country simply by adding each of the disease modules together as needed. Mapping needs within a country may differ by region or district, which can easily be accommodated within the CTM.

**Table 1.** Advantages and disadvantages of disease-specific prevalence and coordinated threshoid mapping

	Disease-specific prevalence mapping	Coordinated threshold mapping (CTM)
**Advantages**	Workers in each disease programme are accustomed to working independently, managing their own budgets and personnel Diseases differ in their geographical distribution; some are more localised (schistosomiasis, onchocerciasis) while others occur widely (trachoma, soil-transmitted helminths, lymphatic filariasis) Produces disease prevalence data for trachoma Reveals whether threshold for public health intervention has been surpassed	Workers share logistical responsibilities, reducing the burden on each programme and allowing them to achieve disease-specific, non-mapping objectives Employs smaller survey teams thus encouraging each team member to perform multiple tasks – saving money and better utilising broadly trained technicians Reveals and improves understanding of occurrence of co-infections among individuals within the population Unites NTD control programmes with respect to public Encourages involvement of community members -building local capacity and local advocacy Reveals whether threshold for public health intervention has been surpassed
**Disadvantages**	Mapping efforts must be mobilised independently Each NTD control coordinator has responsibility of disease mapping	Does not produce precise prevalence data for trachoma Increases responsibility of all survey team members

Next, a CTM team is formed, including: one supervisor (usually selected from among the national-level NTD-control programme coordinators), and one or two diagnostic technicians per NTD to be mapped. The team members are selected for their demonstrated expertise and independence in NTD diagnosis, as well as for their prior field experience and enthusiasm for collaboration. Team members will be trained in CTM methodology, sampling, questionnaires, data collection tools, and in obtaining informed consent.

The CTM team (supervisor and diagnostic technicians) travels in one vehicle to survey two villages in each subdistrict each day. Depending on which diseases are being mapped, some combination of the following activities takes place: trachoma examinations, stool collection, urinalysis, and LF testing (during the day) as well as examination of stool samples (Kato-Katz method) in the evening. Community health workers, teachers, and village chiefs are encouraged by the NTD control programme coordinators to assist with bringing participants to a central location, then organising and registering them. This participation of local volunteers has been shown to build local capacity and spontaneous local advocacy for co-ordinated NTD control.^6^

Co-ordinated (or integrated) mapping surveys have been successfully implemented in several countries, including for schistosomiasis and trachoma in Nigeria^7^, forschistosomiasis, STH and trachoma in Togo^6^; for LF, loiasis, schistosomiasis and STH in South Sudan^8^, ^9^, for trachoma, LF, schistosomiasis and STH in Mali and Senegal^1^; and forschistosomiasis and STH in Cameroon.^10^

Although CTM offers several important advantages over the WHO's disease-specific protocol for countries that are endemic for more than one NTD, the method is not without shortcomings (Table 1). However, the various NTD programmes share the common goal of a healthier, more productive population. By working together in disease mapping, this common goal is more attainable. In our experience, the initial reluctance towards collaboration among different NTD programmes was eased as members quickly noticed improved efficiency compared to disease-specific methodologies.^1^

**Table 2.** Diagnostic methods thresholds used in coordinated threshold mapping (CTM). These are the same as those recommended by the WHO, except for trachoma: the protocol for this disease was adapted in collaboration with the International Trachoma Initiative

**Disease**	**Diagnostic method**	**Thresholds for different public health interventions**
**Trachoma^2^**	Clinical examination using the WHO Simplified Trachoma Grading System	Follicular trachoma (TF) present in >10% of children examined (1-9 years old)
		TF in >5 of children examined (1-9 years old)
		Trichiasis present in >1% of adults (>15 years old)
**Onchocerciasis^3^**	The prevalence of nodules is determined via the rapid epidemiological mapping method (REMO); **or** the presence of *Onchocerca volvulus* microfilaria is determined via skin snip	Forcontrol: presence of palpable nodules in >20% of adults tested (>15 years old)
		For elimination: prevalence of palpable nodules in >5% of adults tested (>15 years old)
**Lymphatic filariasis(LF)^4^**	Immuno-chromatographic card test (ICT) to determine the presence of daytime antigenemia	Present in >1% of adults tested (>15 years old)
**Soil-transmitted helminths (STH)^5^**	Kato-Katz method to look for presence of eggs in stool	Present in >50% of children tested (5-14 years old)
		Present in >20% **and** <50% of children tested (aged 5-14 years)
**Schistosomiasis^5^**	***Schistosoma mansoni:***Kato-Katz method to look for presence of eggs in stool.	Present in >50% of children tested (aged 5-14 years) if based on parasitological methods; **or** >30% if based on questionnaires for visible haematuria
	***Schistosoma haematobium:***Reagent strips (urinalysis) to look for blood in urine and administer questionnaire **or** urine filtration to look for eggs in urine	Present in >10% **and** <50% if parasitological methods; **or** > 1% **and** <30% if based on questionnaire forvisible haematuria in children aged 5-14 years
		Present in >1% **and** <10% (if based on parasitological methods) in children aged 5-14 years
